# Retinal inner nuclear layer thickness in the diagnosis of cognitive impairment explored using a C57BL/6J mouse model

**DOI:** 10.1038/s41598-023-35229-x

**Published:** 2023-05-19

**Authors:** Jack J. Maran, Moradeke M. Adesina, Colin R. Green, Andrea Kwakowsky, Odunayo O. Mugisho

**Affiliations:** 1 Buchanan Ocular Therapeutics Unit, Department of Ophthalmology and The New Zealand National Eye Centre, University of Auckland, Auckland, New Zealand; 2 Department of Ophthalmology and The New Zealand National Eye Centre, Faculty of Medical and Health Sciences, University of Auckland, Private Bag 92019, Auckland, New Zealand; 3 Centre for Brain Research, University of Auckland, Auckland, New Zealand; 4 Department of Anatomy and Medical Imaging, University of Auckland, Auckland, New Zealand; 5 Pharmacology and Therapeutics, School of Medicine, Galway Neuroscience Centre, University of Galway, Galway, Ireland

**Keywords:** Retina, Cognitive ageing

## Abstract

Major neurocognitive disorder (NCD) affects over 55 million people worldwide and is characterized by cognitive impairment (CI). This study aimed to develop a non-invasive diagnostic test for CI based upon retinal thickness measurements explored in a mouse model. Discrimination indices and retinal layer thickness of healthy C57BL/6J mice were quantified through a novel object recognition test (NORT) and ocular coherence tomography (OCT), respectively. Based on criteria from the *Diagnostic and statistical manual of mental disorders 5th ed.* (DSM-V), a diagnostic test was generated by transforming data into rolling monthly averages and categorizing mice into those with and without CI and those with a high or low decline in retinal layer thickness. Only inner nuclear layer thickness had a statistically significant relationship with discrimination indices. Furthermore, our diagnostic test was 85.71% sensitive and 100% specific for diagnosing CI, with a positive predictive value of 100%. These findings have potential clinical implications for the early diagnosis of CI in NCD. However, further investigation in comorbid mice and humans is warranted.

## Introduction

Neurocognitive disorder (NCD), formerly known as dementia, is characterized by a cluster of acute and chronic neurological degenerative diseases, including Alzheimer’s disease (AD), Parkinson’s disease (PD), Parkinson’s disease dementia (PDD), dementia with Lewy bodies (DLB), frontotemporal dementia (FTD), vascular dementia, and acute delirium. The American Psychiatric Association specifically subdivides NCD into major and minor NCD (previously known as dementia and mild cognitive impairment, respectively) in the *Diagnostic and statistical manual of disorders 5th ed.* (DSM-V), where both involve a substantial decline from baseline in cognitive function in at least one of the following six cognitive domains: complex attention, executive ability, learning and memory, language, motor and visual perception, and social cognition^1^. The DSM-V further characterizes major NCD as involving an appropriate neurocognitive test score two or more standard deviations below the mean. Similarly, minor NCD is defined by a test score between one and two standard deviations below the mean^1^.

Globally, major NCD is the seventh leading cause of death and represents a significant public health burden that costs the global economy over US$1 trillion annually, with over 55 million people who currently suffer from this debilitating condition^2,3^. Notably, the 2020 *Lancet* commission identified 12 main risk factors that contribute to the development of NCD, namely: hypertension, hearing impairment, a lower level of education, smoking, obesity, depression, physical inactivity, diabetes, low social contact, excess alcohol consumption, traumatic brain injury and air pollution^2^.

While the risk factors for the development of dementia are relatively well-elucidated, diagnosis and management of dementia pose many challenges. Predominantly, the diagnosis of dementia is unobjective and guided by the subjective clinical suspicion of a patient’s primary care physician or family members who have noticed a gradual decline in an individual’s cognitive function. These issues pose a challenging predicament with accurate diagnoses of cognitive impairment (CI) in NCD, particularly in patients with early-onset cognitive decline, concurrent depression or delirium, language barriers, differences in the baseline level of education, or older adults who sparingly interact with the healthcare system^4–7^. As such, accurate and timely diagnosis of CI during NCD is limited due to overwhelming confounding and complicating factors that may mask objective diagnosis by clinicians. A notable review of 40 studies by Bradford et al. indicated that the diagnostic sensitivity of NCD by primary care physicians based on DSM-V criteria ranged from 0.26 to 0.60 and was as poor as 0.09 in patients with mild NCD^4^. Moreover, neuropsychological tests are not available in all settings and thresholds for tests are affected by various factors such as sensory limitations, systemic comorbidities, and the test environment^4,6^.

Given the difficulty with accurately diagnosing CI during mild and major NCD, patients frequently experience delayed diagnoses, where an accurate diagnosis can only be made after significant disease progression^4^. Moreover, patients may also be incorrectly referred to clinicians for investigation of NCD for subjective or subclinical symptoms, which places further unnecessary strain on the healthcare system due to increased patient loads and inappropriate referrals for patients who are investigated for NCD but do not have NCD.

The limitations highlighted above indicate the need for a simple, inexpensive, and out-of-hospital diagnostic test for CI in NCD that can objectively monitor the neural environment at various time points throughout an individual’s life. Several studies have suggested that the retina is an anatomical extension of the brain and that pathological changes in the brain are often mirrored in the retina, particularly during neurocognitive diseases^8,9^. For example, during AD, amyloid-β deposition may occur within multiple retinal layers, along with retinal thinning, retinal ganglion cell death microglial infiltration and astrogliosis^10–14^. Furthermore, phosphorylated alpha-synuclein may accumulate in the retina in parallel with deposition in the human brain during PD, even during the asymptomatic and preclinical disease stages^15,16^. Even during mild cognitive impairment, retinal microvascular alterations may be detected, further emphasising the interconnectivity between the retina and the brain^17^. Alongside this, one of the most cost efficient, non-invasive and widely used technologies to study the retina is ocular coherence tomography (OCT), which is readily available for ophthalmic assessment^18^.

Moreover, previous studies in mice and humans have shown that the thickness of various retinal layers may be decreased in patients diagnosed with AD, FTD and PD^19–23^. However, few studies have adequately investigated the association between the thickness of retinal layers with CI in a cognitively healthy population at baseline^24,25^. Therefore, this study investigates the effectiveness of retinal layer thickness measured by OCT in diagnosing cognitive impairment, utilizing a C57BL/6J mouse model.

## Results

### Retinal layer thickness has a positive linear relationship with discrimination index

**Figure 1:**
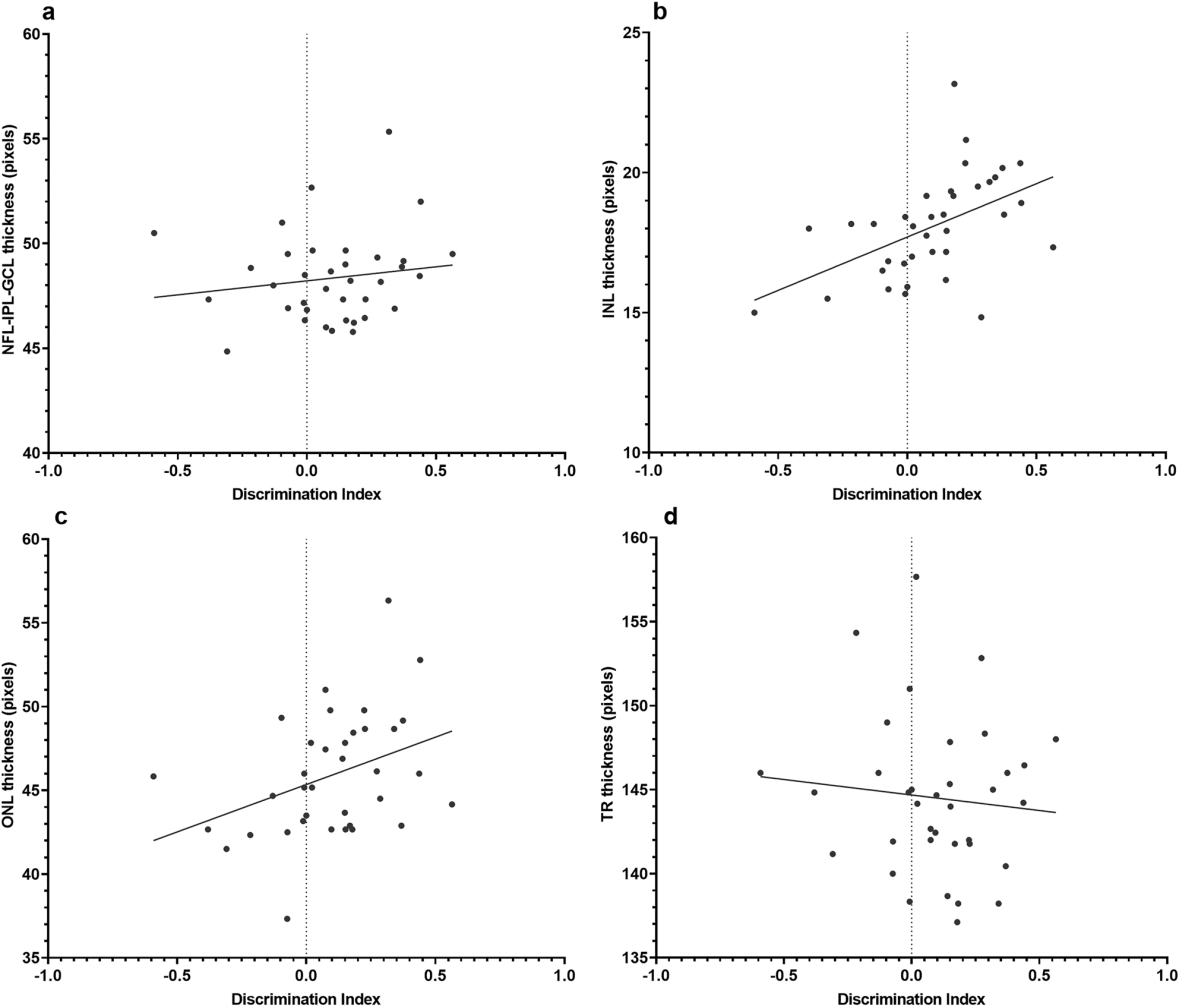
Linear regression between absolute discrimination indices quantified by NORT and retinal layer thickness measured by OCT in 4-month-old C57BL/6J mice at baseline, 6 months and 9 months of age. Relationships were considered significant if the gradient of regression lines were significantly non-zero. Only INL and ONL data demonstrated significant relationships. (**a**) There is a non-significant positive linear relationship between absolute NFL-GCL-IPL thickness and discrimination index (p = 0.3826). (**b**) There is a significant positive linear relationship between INL thickness and discrimination index (p = 0.0020). (**c**) There is a significant positive linear relationship between ONL thickness and discrimination index (p = 0.0264). (**d**) There is a non-significant negative relationship between TR thickness and discrimination index (p = 0.5785). *NFL-GCL-IPL* nerve fibre layer-ganglion cell layer-inner plexiform layer, *INL* inner nuclear layer, *ONL* outer nuclear layer, *TR* total retina thickness. NFL-GCL-IPL, INL and ONL positively correlated with discrimination indices, whereas TR was negatively correlated with discrimination indices. Data points represent one mouse for each eye at a single point in time. Regression line equations and r^2^ values for each dataset are presented in Supplementary Table S1.

When retinal layer thickness was analyzed against discrimination index, mice demonstrated a positive linear relationship with discrimination index in all measured retinal layers (Fig. 1). However, the gradient of the linear relationship was only significantly non-zero in the ONL and INL layers (Supplementary Table S1).

**Figure 2:**
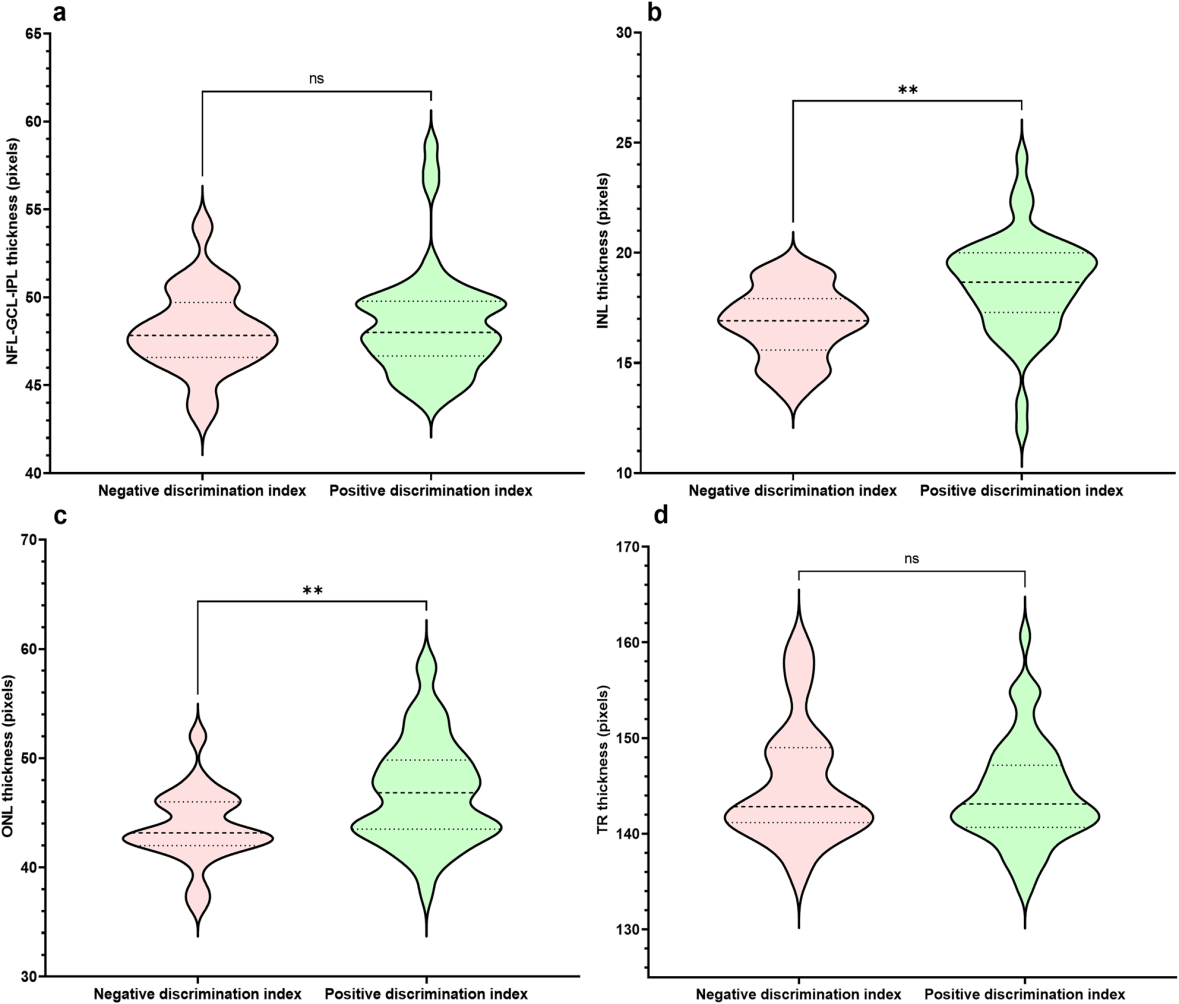
Violin plots of retinal layer thickness in 4-month-old C57BL/6J mice at baseline, 6 months and 9 months of age, grouped according to positive or negative discrimination index. Data were first analyzed with an F test for equal variances. Normality of residuals was also assessed by Anderson–Darling, D’Agostino-Pearson omnibus, Shapiro–Wilk, and Kolmogorov–Smirnov tests. INL and ONL data passed all normality and equal variance tests and were assessed for differences between groups with two-tailed unpaired t-tests. NFL-GCL-IPL and TR data failed all normality tests and were assessed for differences between groups with Mann–Whitney U tests. (**a**) There is no significant difference between NFL-GCL-IPL thickness between mice with positive and negative discrimination indices (p = 0.8340). (**b**) INL thickness is significantly increased in mice with positive discrimination indices than in those with negative discrimination indices (p = 0.0010). (**c**) ONL thickness is significantly increased in mice with positive discrimination indices than in those with negative discrimination indices (p = 0.0053). (**d**) There is no significant difference between TR thickness between mice with positive and negative discrimination indices (p = 0.6910). *NFL-GCL-IPL* nerve fibre layer-ganglion cell layer-inner plexiform layer, *INL* inner nuclear layer, *ONL* outer nuclear layer, *TR* total retina thickness, *Ns* not significant; **p < 0.01.

Likewise, when mice were grouped according to negative or positive discrimination indices, statistical analysis revealed significant increases in the INL (1.817 ± 0.5291, *p* = 0.0010) and ONL (3.179 ± 1.105, *p* = 0.0053) thickness in mice with positive discrimination indices compared to negative discrimination indices (Fig. 2). No significant differences were observed in the NFL-GCL-IPL (p = 0.8340) or TR groups (p = 0.6910).

### NFL-GCL-IPL thickness is a significant predictor of total retinal thickness

Previous studies have mainly investigated NFL and TR thickness in relation to cognitive function^24,26–30^. However, TR thickness is a compound measure that already includes NFL-GCL-IPL, INL and ONL thicknesses, which can lead to collinearity and statistical bias. As such, NFL-GCL-IPL, INL, and ONL thicknesses were analyzed against TR thickness with multiple linear regression. This was done to determine if TR thickness was significantly predicted by another retinal layer while adjusting for confounding between layers.

Multiple linear regression of raw NFL-GCL-IPL, INL, ONL and total retinal (TR) data showed that NFL-GCL-IPL data was a significant predictor of TR thickness (β = 1.257, p = 0.0014) (Supplementary Table S2). Furthermore, this trend was consistent when data was transformed into a rolling monthly change where the absolute monthly change in NFL-GCL-TR thickness was a significant predictor of the absolute monthly change in TR thickness (β = 1.501, p = 0.0007) (Supplementary Table S3). Therefore, TR thickness was not utilized as a metric to evaluate discrimination index, as NFL-GCL-IPL thickness was already modelling TR thickness satisfactorily.

### The absolute monthly change in INL thickness is a significant predictor of the monthly change in discrimination index

Multiple linear regression analysis showed that the absolute monthly change in INL thickness had a significant relationship with the monthly change in discrimination index (β2 = 0.0982, p = 0.0123) (Table 1). Analysis for collinearity revealed that the correlation between variables was all below 0.8, indicating minimal collinearity between NFL-GCL-IPL, INL and ONL thickness (Table 1). Analysis of the overall regression model was also significant, where the regression Omnibus test was statistically significant (p = 0.0420) (Table 2). As such, the linear regression model utilized was appropriate for predicting the discrimination index in mice. The overall regression multiple R, r^2^, and adjusted r^2^ values were 0.5745, 0.3301 and 0.2296, respectively.

**Table 1:** Multiple linear regression coefficients and covariance matrix for the absolute monthly change in discrimination index and retinal thickness.

Multiple linear regression coefficients				
Layer	Regression coefficient	P-value	VIF	r^2^ with other variables
Intercept	0.0266	0.5417	–	–
NFL-GCL-IPL	− 0.0072	0.8030	1.225	0.1839
INL	0.0982	0.0123*	1.186	0.1565
ONL	0.0171	0.3333	1.053	0.0504

Parameter covariance correlation matrix				
	β0	β1	β2	β3
β0	1.0000			
β1	0.2150	1.0000		
β2	0.6273	0.3774	1.0000	
β3	0.3934	− 0.1859	0.0465	1.0000

Statistical analysis was carried out using GraphPad Prism 9.3.0 software. Data were analyzed by multiple linear regression. VIF: variance inflation factor. *p < 0.05; **p < 0.01. β denotes the coefficients of variables in the multiple linear regression equation. β0: intercept, β1: NFL-GCL-IPL, β2: INL, β3: ONL. Values in the correlation matrix represent regression coefficients between variables.

**Table 2:** Overall multiple regression model analysis of variance.

	SS	DF	MS	F (DFn, DFd)	P-value
Regression	0.2049	3	0.06832	F (3, 20) = 3.285	0.0420*
NFL-GCL-IPL	0.0013	1	0.00133	F (1, 20) = 0.0639	0.8030
INL	0.1577	1	0.1577	F (1, 20) = 7.5810	0.0123*
ONL	0.0204	1	0.0204	F (1, 20) = 0.9831	0.3333
Residual	0.4159	20	0.0208		
Total	0.6209	23			

*SS* sum of squares, *DF* degrees of freedom, *MS* mean square, *F* F-ratio, *DFn* degrees of freedom (numerator), *DFd* degrees of freedom (denominator).

Interestingly, the absolute monthly changes in NFL-GCL-IPL and ONL thickness were not found to have a significant linear relationship with discrimination index (p = 0.8030 and 0.3333, respectively).

### There is a positive linear relationship between absolute monthly change in INL thickness and discrimination index

**Figure 3:**
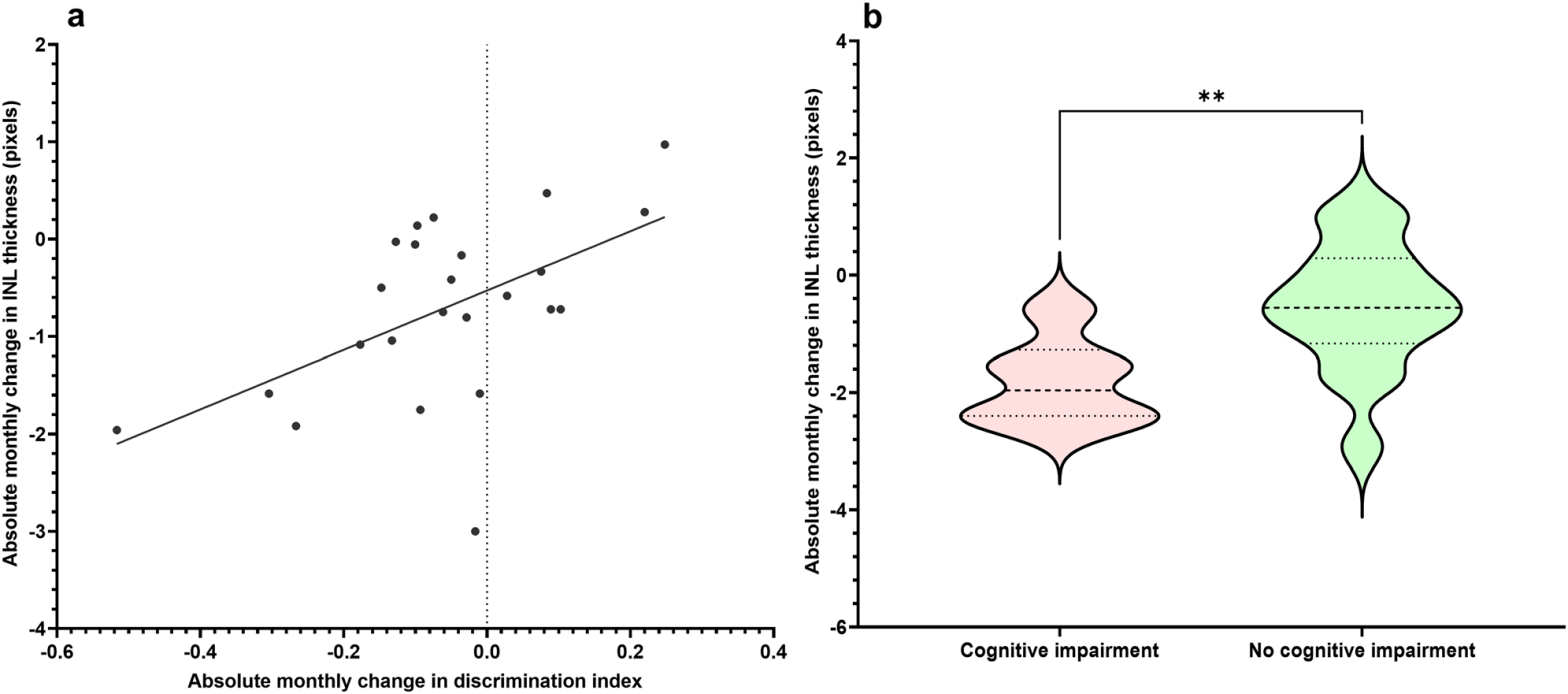
(**a**) Linear regression between absolute monthly change in discrimination index and retinal layer thickness measured by OCT in 4-month-old C57BL/6J mice. Discrimination indices were quantified by a NORT at 4 months of age, 6 months of age and 9 months of age. The absolute monthly change in discrimination indices was calculated by the difference in discrimination indices divided by the time elapsed between the measurements in 4, 6, and 9 months. Absolute monthly changes in retinal layer thickness were calculated similarly. Relationships were considered significant if the gradient of regression lines were significantly non-zero. There is a significant positive linear relationship between the absolute monthly change in INL thickness and the absolute monthly change in discrimination index (y = 3.046x − 0.5284, r^2^ = 0.2970, p = 0.0059). (**b**) Violin plot of the absolute monthly change in retinal layer thickness in C57BL/6J mice with and without NCD. Based on the DSM-V, the threshold of NCD was set as an absolute monthly change in discrimination index less than one standard deviation below the mean, which was − 0.2222 (µ = − 0.0579, σ = 0.1643). Mice below this threshold were considered to have NCD, and mice above this threshold were considered to have no NCD. Data were first analyzed with an F test for equal variances. Normality of residuals was determined through Anderson–Darling, D’Agostino-Pearson omnibus, Shapiro–Wilk, and Kolmogorov–Smirnov tests. INL data passed all normality and equal variance tests and were assessed with two-tailed unpaired t-tests. Absolute monthly change in INL thickness was significantly increased in mice without NCD compared to mice with NCD (p = 0.0083). *INL* inner nuclear layer, *ONL* outer nuclear layer. ** p < 0.01.

As shown in “The absolute monthly change in INL thickness is a significant predictor of the monthly change in discrimination index”, the absolute change in INL thickness was the most strongly associated with cognitive impairment after controlling for changes in the thickness of the other layers. Furthermore, the absolute change in NFL-GCL-IPL and ONL thickness did not have a significant linear relationship with discrimination index. As such, the absolute change in INL thickness was selected as the most sensitive reflection of discrimination index. In order to individually assess the effect of discrimination index on inner nuclear layer thickness, simple linear regression was conducted, which showed that the absolute change in monthly discrimination index and INL thickness had a positive linear relationship (r^2^ = 0.2970) (Fig. 3A). The gradient of the respective regression line was significantly non-zero as well ($y=3.046x-0.5284$, *p* = 0.0059). The mean change in the monthly discrimination index was − 0.0579, and the standard deviation was 0.1643. Therefore, the threshold for CI was − 0.2222 per month.

**Figure 4:**
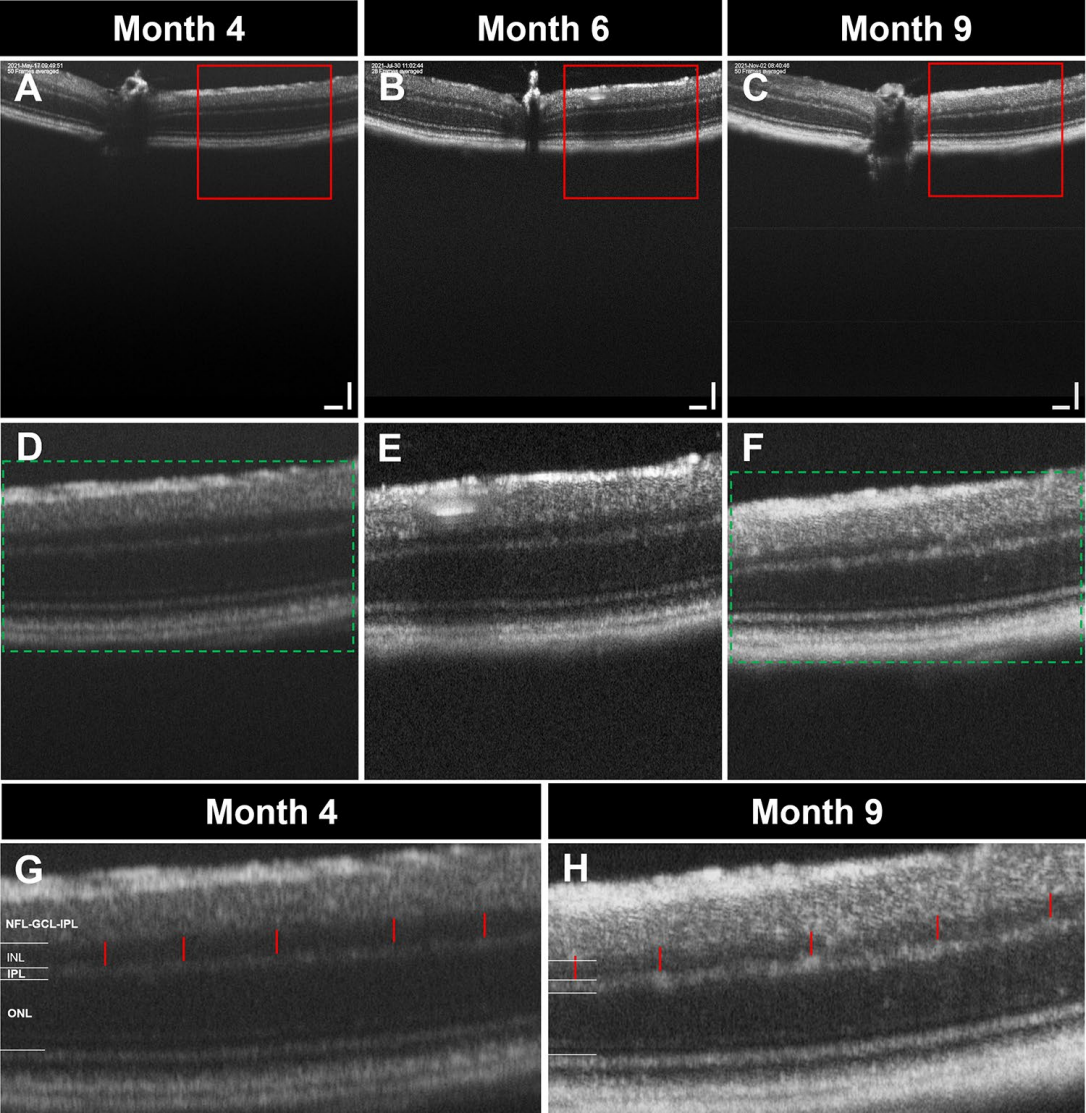
Serial representative OCT images of mice retina over time. Distinct thinning of the INL was prominent with time. Particularly, the most significant thinning was noted between 4 months of age and 9 months of age. Red boxes and green rectangles denote regions of interest that were zoomed in upon in subsequent images. (**A**–**C**) OCT images of a mouse at 4 months, 6 months and 9 months of age respectively. (**D**–**F**) Zoomed in OCT images of red boxes indicating clear INL thinning. (**G**,**H**) Zoomed in OCT images of green boxes. Red vertical lines represent equidistant lines which have been overlayed onto the INL, showing clear INL thinning between 9 months of age and 4 months of age.

Moreover, further statistical analysis revealed that mice without CI had significantly greater monthly INL preservation (1.274 ± 0.4620, *p* = 0.0083) compared to mice with NCD (Fig. 3B). No significant differences were identified in the ONL group (p = 0.3469). Furthermore, qualitative analysis of OCT images obtained from mice at 4 months, 6 months, and 9 months of age showed a clear thinning of the INL with time (Fig. 4), which further supports statistical findings indicating that INL thickness is associated with cognitive impairment.

### The absolute monthly change in discrimination index is highly sensitive and specific for diagnosing CI in mice

**Figure 5:**
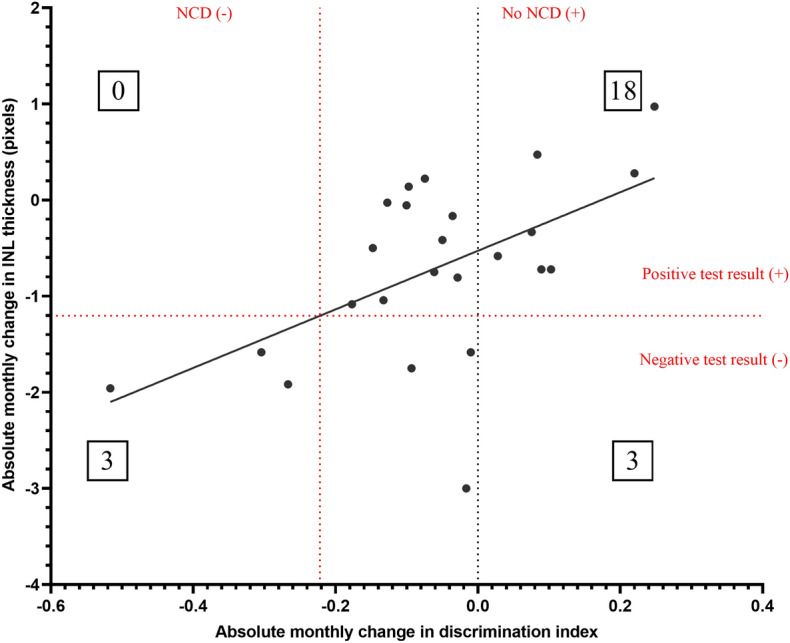
Combined contingency table and linear regression between absolute monthly change in discrimination index and retinal layer thickness measured by OCT in 4-month-old C57BL/6J mice. Discrimination indices were quantified by a NORT at 4 months of age, 6 months of age and 9 months of age. The absolute monthly change in discrimination indices was calculated by the difference in discrimination indices divided by the time elapsed between the measurements in 9 m vs 6 m and 6 m vs 4 m of age. Based on the DSM-V, the threshold of NCD was set as an absolute monthly change in discrimination index less than one standard deviation below the mean, which was − 0.2222. Mice below this threshold were considered to have NCD, and mice above this threshold were considered to have no NCD. The threshold for the absolute change in INL thickness for the diagnostic test was interpolated using the INL regression line equation in Fig. 3 (y = 3.046x − 0.5284), which was − 1.205 pixels per month. Mice above this threshold for the absolute change in INL thickness were considered to have a positive diagnostic test outcome, and mice below this threshold had a negative test outcome. 21 of 24 mice had NCD, while 3 of 24 mice had no NCD. All mice with NCD had a negative test outcome. 18 of 21 mice without NCD had a positive test outcome. None of the mice with a negative test outcome had an absolute monthly change in discrimination index higher than zero. The contingency table was analyzed with a Fisher’s exact test for statistical significance (p = 0.0099**). Sensitivity = 0.8571, specificity = 1.0000, positive predictive value = 1.0000, negative predictive value = 0.5000. Data points represent values for one mouse at either six months vs baseline or 9 months vs baseline. INL = inner nuclear layer.

The threshold for the absolute monthly change in INL thickness was determined to be − 1.205 pixels per month based on interpolation of the CI threshold of − 0.2222 into the INL regression equation in Fig. 2A (y = 3.046x − 0.5284). These threshold values allowed for developing a diagnostic test, where 21 of 24 data points were classified as not having CI (Fig. 5). The population prevalence of CI in this study was correspondingly 3 in 24 (12.50%). 18 of 21 data points without CI had a positive test outcome, whereas all 3 of 3 data points with CI had a negative test outcome.

The contingency table in Fig. 3 was further analyzed with a Fisher’s exact test and was statistically significant (*p* = 0.0099). Predictive analysis revealed that the diagnostic test was highly sensitive (85.71%) and specific (100.00%), with an LR and DOR of infinity. PPV and NPV were 100.00% and 50.00%, respectively, for the population CI prevalence of 12.5% examined in this study. Further predictive parameters, as well as evaluative parameters of the CI diagnostic test, are fully summarised below in Table 3.

**Table 3:** Basic diagnostic test parameters and evaluative parameters of CI diagnostic test.

Basic diagnostic test parameters	
Predictive parameters	Value
p-value (two-tailed Fisher’s exact test)	0.0099
Sensitivity	0.8571
Specificity	1.0000
Positive predictive value (PPV)	1.0000
Negative predictive value (NPV)	0.5000
Relative risk (RR)	2.0000
Diagnostic odds ratio (DOR)	Infinity
Likelihood ratio (LR)	Infinity

Evaluative parameters of CI diagnostic test	
Evaluative parameters	Value
Youden’s Index (J)	0.8571
NND	1.1667
NNM	8.0000
PSI	0.5000
Inverse of PSI	2.0000

## Discussion

In the present study, a diagnostic test for neurocognitive disorders was devised, where the monthly changes in INL thickness and discrimination indices of C57BL/6J mice were concurrently measured over time. Through predictive analysis, our data demonstrated that the absolute monthly change in INL thickness strongly reflects the change in discrimination indices in C57BL/6J mice. Moreover, a positive linear relationship between the absolute monthly change in INL thickness and discrimination index was identified, where a high level of monthly decrease in INL thickness more significant than the specified threshold was associated with CI. Notably, individuals with positive cognitive test results were extremely likely to have no CI.

This diagnostic test demonstrated a high sensitivity of 85.71% and specificity of 100.00%, indicating very high effectiveness in diagnosing CI. Additionally, further evaluation yielded a high Youden’s index of 0.8571, NNM of 8.000 and a low NND of 1.1667, which indicated high-test efficacy in populations irrespective of population prevalence of CI. Particularly, an NND of 1.1667 indicated a high success rate, where only one subject must be investigated to diagnose one person with CI correctly, irrespective of the population disease prevalence. Evaluation of this test with NNP yielded a value of 2.000 due to being impacted by this study’s low population prevalence of CI (12.50%), as the majority of the test subjects were healthy. However, if the prevalence of CI were closer to 50.00% in a population assessed by this diagnostic test, this value would likely be more optimistic.

Remarkably, the diagnostic test demonstrated a very high positive predictive value (100.00%), where if a test subject had a positive test result, there was a 100.00% probability of having cognitive impairment. Moreover, this is supported by the test’s excellent DOR and LR of infinity, indicating high diagnostic utility with positive test results. These values indicate that this test is an excellent predictor of cognitive health and may be utilized as a ruling-out test for cognitive impairment in mice^31^. Although the negative predictive value was low at 50.00%, it is crucial to note that none of the mice which had a negative test had a monthly change in cognitive impairment that was above zero, which indicates that a negative test result may still be clinically significant and provide enough reason for further cognitive investigation to diagnose CI accurately. A study by Davis et al. of 18,103 patients over the age of 65 showed that there is a continuum of progression from normal cognition to mild cognitive impairment to Alzheimer’s disease, where 8% of participants with normal cognition at baseline progressed to at least mild cognitive impairment within a year^32^. With relevance to our data, although the NPV was 50% for detecting CI, individuals with a negative test who do not have cognitive impairment may represent patients at risk of progressing into mild cognitive impairment over time. A potential clinical application would be greater surveillance and cognitive testing over time for patients with negative test results. Furthermore, if this diagnostic test was applied in a primarily diseased population, the NPV might be significantly higher and carry a much greater clinical utility as a ruling-in test for CI.

Overall, when utilized in a population with low disease prevalence, this diagnostic test may be best utilized as a test of health where CI is ruled out due to the very optimistic PPV. However, if this test were applied to a more diseased population, the NPV would be much higher, and the test would carry more utility as a ruling-in test for CI instead. Therefore, depending on the population prevalence of CI and an individual’s pre-test probability, this test may be utilized as both a ruling-in and ruling-out diagnostic test.

While this study has suggested that INL thickness may be used to predict CI in mice, the underlying pathophysiological link between the retinal layer thickness and cognitive function remains unknown. In fact, most previous studies have suggested that thinning of the RNFL or GCL-IPL complex may be associated with cognitive decline^24,26–30^. However, our findings indicated that only the INL thickness was the most reliable predictor of cognitive function, whereas the NFL-GCL-IPL and the ONL thicknesses showed no significant relationship with discrimination indices in mice. One potential reason for this could be that the NFL, GCL and IPL layers were reported as a single measure in these mice due to the inability to distinguish between these layers on OCT accurately. However, it is important to note that most studies have suggested that thinning of the inner retina was the most associated with cognitive decline, and very few studies have suggested that the outer retina has a link to CI.

Furthermore, Lad et al. identified that there might instead be a thickening of the NFL and GCL-IPL layers during AD progression, possibly due to retinal gliosis preceding neuronal loss^33^. Other studies have also reported this paradoxical pattern, which have suggested that NFL thickness may have an inverse relationship with cognitive function during MCI^34^. Indeed, various investigations have suggested that a decline in NFL and GCL-IPL thickness was more associated with advanced dementia, but in the early stages of dementia or preclinical disease, the NFL and GCL-IPL may be thickened instead^33,35,36^. Given that we examined a population of mice without established neurodegenerative diosrder at baseline, this may explain why no significant changes were observed in the NFL-GCL-IPL.

Another possible mechanism linking INL thickness and CI may be attributable to the effect of α-synuclein on dopaminergic neurons within the INL and hippocampus, which promotes inflammasome-mediated chronic low-grade inflammation in both the INL and the brain^37–39^. α-synuclein has been well-established to be neurotoxic and cause local dopamine depletion by toxicity to dopaminergic cells in the central nervous system and inhibits dopamine transport and synthesis^40–45^. Recent research has suggested that α-synuclein accumulation in the brain may play a central role in the pathogenesis of many neurocognitive disorders, including PD, PDD, DLB and AD^40,43–52^. Moreover, in AD, amyloid-β may also trigger the accumulation of α-synuclein aggregates in the brain and the retina^45,48,50,53,54^.

Additionally, studies have further elicited that α-synuclein accumulates mainly in the inner retina in humans with CI^42,49,55^. Although we did not directly assess the localization of α-synuclein within the retina of the mice in this study, previous studies by Martinez-Navarrete et al. showed that there was a prominent aggregation of α-synuclein within the inner retina in vertebrates, including mice, rats, rabbits and cows. Of note, α-synuclein was found to accumulate around amacrine cells (including dopaminergic amacrine cells) in *Mus musculus* C57BL6/J mice, which are anatomically located at the boundary between the INL and IPL^56^. Moreover, this pattern of aggregation has also been demonstrated in healthy humans without CI, where α-synuclein was located mainly in the INL in the majority of cases^56,57^. Taken together, dopamine concentrations may be depleted in the INL in NCD due to the accumulation of α-synuclein in the inner retina, particularly around dopaminergic amacrine cells^40,42,58^.

With relevance to the scope of this study, dopamine may have neuroprotective effects, such as upregulating the cAMP/PKA pathway, preventing NMDA-induced neuroapoptosis and inhibition of the pro-inflammatory nod-like receptor protein-3 (NLRP3) inflammasome pathway^37,59–69^. Some studies have also suggested that reduced dopamine levels may deplete substance P levels, which is protective against inner retinal inflammation, apoptosis, and VEGF-induced vascular breakdown^50,70–75^. Importantly, studies have shown that molecular changes in the brain may occur in the retina simultaneously, where α-synuclein is deposited in the brain and retina at the same time during the pathogenesis of neurocognitive disorder^9,26,30,42,48,50,76^. As such, along with the physiological location of dopaminergic amacrine cells in the brain and the retina, the idea that α-synucleinopathy and dopamine dysfunction are early and common pathways in neurocognitive disorders may explain why INL may be such a sensitive predictor of cognitive decline.

An important application of these findings is the early detection of cognitive decline through a non-invasive simple test. Often, dementia is preceded by several years of asymptomatic cognitive decline^77,78^. Using OCT to gauge cognitive decline in a repeatable non-invasive manner may allow for monitoring and early diagnosis of major neurocognitive disorders prior to the onset of clinically observable disease features. Furthermore, this may allow for earlier treatment and prevention strategies to be enacted, which can reduce an individual’s risk of developing cognitive disorders in the future.

In order to adequately apply these findings to a clinical context, an important consideration is that mice in this study did not have any concurrent retinal degenerative diseases such as diabetic retinopathy or age-related macular degeneration (AMD). However, in clinical practice with humans, subjects may likely have concurrent retinal diseases that may affect the validity of this diagnostic test. As such, further research is crucial, and this experiment should be repeated with mice with specific retinal conditions, such as AMD or diabetic retinopathy. Moreover, this study was not conducted in mice of varying ages at baseline. In order to account for these potential differences at baseline, it is critical to repeat this study in mice with different ages and retinal diseases, where individual diagnostic contingency tables may be developed for each of these confounding factors. At the same time, previous research has demonstrated that AMD and DR significantly correlate with reduced cognitive test scores^79–81^. AMD has also been correlated with major NCD diagnoses, particularly AD^79,80^. Therefore, although AMD and DR may affect thickness measurements of the retina, with further research, it is likely that this diagnostic test will still maintain fidelity even in patients with concurrent retinal diseases and demonstrate a similar pattern to that which was observed in this study^82^.

Another consideration that must be made is the assumption that test subjects who have cognitive impairment are experiencing an active process of neuroinflammation in both their brain and retina at the same time and have not lost neuronal tissue to the extent that the decline is no longer linear over time. In the case of a test subject with a very advanced neurocognitive disease, there may be a low level of monthly decline in cognitive function and INL thickness due to having a substantially lower baseline in the first place. Nevertheless, if this scenario were applied to actual clinical practice, these individuals would have already been identified by their general practitioner or neurologist as having NCD due to having a pre-existing poor baseline. In these circumstances, an ocular diagnostic test for NCD will not have been necessary in the first place.

## Conclusion

In conclusion, the absolute monthly change in INL thickness measured by OCT appears to be a highly sensitive and specific diagnostic test for cognitive impairment in C57BL/6J mice. However, further investigations in mice with concurrent retinal diseases and humans are required before translating this to clinical practice. With further research, this diagnostic marker could become an accurate, accessible, non-invasive, and inexpensive diagnostic test for NCD, thereby streamlining referrals of patients to specialists for tertiary treatment.

## Material and methods

### Animal husbandry

All experiments were conducted with ethics approval and in accordance with the regulations of the University of Auckland Animal Ethics Committee (approval number: AEC3369) and the ARRIVE guidelines for animal research. Twelve C57BL/6J male mice aged 4 months without any known mutations, ocular disease and systemic pathology were housed under standard laboratory conditions between 22 and 23 °C at the Vernon Jansen Unit (VJU) Animal Facility, University of Auckland. Mice were fed with Prolab® RMH 1800 (LabDiet; USA) and had access to water and food ad libitum. A twelve-hour light–dark cycle was maintained throughout housing.

### Deriving the discrimination index

Mice were subjected to a novel object recognition test (NORT) according to the protocol previously described by Yeung et. al.^83^ and Calvo-Flores Guzmán et al.^84^. Mice were tested at 4 months, 6 months, and 9 months of age. NORT was performed in a square arena (26.5 cm × 26.5 cm × 26.5 cm) with non-transparent plexiglass walls and was carried out in a dark PC2 laboratory room under a similar temperature and humidity to housing conditions. Arena walls were covered with black paper, and dim lamps were utilized to illuminate the maze to 20 lx to minimize stress on the mice^85^.

Twenty-four hours prior to testing, a familiarisation phase was conducted by allowing each mouse to habituate to the empty arena individually for 5 min. Subsequently, two identical objects were introduced into the area and mice were allowed to explore the familiar objects for an additional 5 min. A camera and MediaRecorder 4.0 software (Noldus; USA) was used to record mice behaviour. Testing was conducted the following day with all the conditions, cameras, lighting, and room set-up kept constant. One familiar and one novel object were placed in the arena, and mice were placed in the arena to explore the objects for 5 min. The arena and objects were thoroughly cleaned with 70% ethanol and aired in the room for 5 min between animals to eliminate olfactory cues.

Videos of mice were analyzed, and discrimination indices were calculated as the difference in time spent with a novel object minus the time spent with a familiar object, divided by each mouse’s total time of exploration in the NORT. Discrimination indices were used as a surrogate measure of long-term memory and cognitive function, where a value above 0 and closer to 1 represented better neurocognitive function in mice.

### Retinal layer thickness quantification

Following the acquisition of NORT data, mice were anaesthetized by intraperitoneal injection of ketamine (50 mg/kg; PhoenixPharm, New Zealand) and medetomidine (0.5 mg/kg; Domitor^®^, Zoetis, New Zealand), and their pupils dilated with 1% tropicamide (Minims, UK) to enable measurement of the thickness of retinal layers with the OCT mode of the Micron IV imaging system (Phoenix Research Labs; USA). Fifty frames were obtained across the retina surrounding the optic nerve head in steps and averaged to form a combined image. Images were analyzed using the ImageJ 1.50i (National Institute of Health, USA). The nerve fibre layer (NFL), ganglion cell layer (GCL) and inner plexiform layer (IPL) were summated and reported as a single measure due to the inability of the OCT to distinguish the boundaries between these layers in mice. Inner nuclear layer (INL) thickness and outer nuclear layer (ONL) thickness were individually reported. Total retinal (TR) thickness was also measured. Following in vivo ocular assessments, mice were awakened by an intraperitoneal injection of atipamezole (5 mg/kg; Antisedan^®^, Zoetis, New Zealand). In the first instance, the thickness of the NFL-GCL-IPL, INL, ONL and layers were segregated into right and left eye data and were analyzed for differences in retinal layer thickness between eyes. Statistical analysis of untransformed data revealed no significant differences in the thickness of retinal layers between the right and left eyes (Supplementary Fig. S4). Therefore, retinal layer thickness in mice was analyzed as an average of both eyes in all further analyses.

### Statistical analysis of discrimination index and retinal layer thickness data

All statistical analyses were conducted using GraphPad Prism 9.3.1 software. All data were assessed for normality of residuals using Anderson–Darling, D’Agostino-Pearson omnibus, Shapiro–Wilk, and Kolmogorov–Smirnov tests. Unpaired data were also additionally assessed with an F-test for equal variances. Only data that were not significant for all tests were considered normally distributed and were therefore assessed with two-tailed t-tests. Data that were not normally distributed were subject to a Mann–Whitney U test. The specific test used for each dataset is provided in the respective figure legends. A corrected *p*-value of less than 0.05 was considered statistically significant in all analyses.

#### Linear relationship between retinal layer thickness and discrimination index

NFL-GCL-IPL, INL, ONL and TR thickness data (as an average of both eyes for each mouse) were plotted against discrimination indices of matched mice and analyzed using linear regression to derive a corresponding trendline r^2^ value. All regression lines were analyzed using GraphPad Prism 9.3.0 to determine if slopes were significantly non-zero.

NFL-GCL-IPL, INL and ONL thickness were segregated based on positive and negative discrimination indices, and differences in retinal layer thickness were assessed between mice using unpaired two-tailed T-tests and Mann–Whitney U tests using GraphPad Prism 9.3.0.

#### Multiple linear regression of absolute monthly changes in both discrimination index and retinal layer thickness

Natural variation and confounders affect the baseline discrimination index, and variation in retinal thickness may exist between each mouse. Therefore, the rolling absolute monthly changes in both the discrimination index and retinal thickness were analyzed. This allowed better isolation of individual trends for each mouse by comparing data for 9 to 6 and 6 to 4-month-old mice. A rolling monthly change in the data was calculated by taking the difference in these numerical values and dividing it by the number of months between the two time periods. This data was utilized in analyzing the relationship between retinal thickness and discrimination index.

In order to account for any confounding between the NFL-GCL-IPL, INL and ONL in relation to discrimination index, rolling monthly changes in discrimination index and retinal thickness were analyzed with a multiple linear regression model to determine the relationship of individual retinal layers with discrimination index. The analysis allowed us to determine which retinal layer had the most statistically significant linear relationship with discrimination index and helped guide the generation of a predictive model. Standardized regression coefficients were calculated for each retinal layer. Coefficients for the absolute monthly change in retinal layer thicknesses with a p-value greater than 0.05 were considered to not significantly influence the overall model.

Retinal layers that were significant during multiple linear regression were analyzed individually against discrimination index with simple linear regression by plotting the absolute monthly change in retinal layer thickness against the absolute monthly change in discrimination index. This is because discrimination index is a surrogate measure of central nervous system health, which likely affects retinal layer thickness.

#### Difference in absolute monthly change in retinal layer thickness between mice with and without neurocognitive disorders

The *DSM-V* defines mild NCD as constituting a neuropsychological test score one to two standard deviations below the population’s mean. Major NCD is similarly defined by a neuropsychological test score more than two standard deviations below the mean^1^. Therefore, the threshold for CI in neurocognitive disorders was set as one standard deviation below the population mean of the absolute monthly change in discrimination index to include CI in both mild and major NCD.

Based on the absolute monthly change in discrimination index data from 2.2.4, one standard deviation below the mean monthly change in discrimination index was − 0.2222 per month. This was set at the threshold for NCD. All mice with changes in absolute discrimination index above − 0.2222 per month were classified as having no NCD, while all mice with values below − 0.2222 were classified as having NCD. The difference in the absolute monthly change in retinal layer thickness between mice with and without NCD was analyzed with unpaired two-tailed t-tests and Mann–Whitney U tests.

#### Predictive analysis of absolute monthly change in retinal thickness and discrimination index

In order to effectively conduct predictive analysis, a similar threshold value for the absolute monthly change in INL thickness was necessary. The threshold for the absolute change in INL thickness was appropriately determined using the INL regression line obtained in 2.2.5. An x-value of − 0.2222 was substituted into the INL regression line equation, y = 3.046x − 0.5284, to derive a corresponding monthly change in the INL thickness threshold of − 1.205 pixels per month.

Therefore, a diagnostic test was generated, where all mice with changes in absolute INL thickness above − 1.205 pixels per month were classified as having a positive test result, indicative of normal cognitive function. All values below − 1.205 pixels were classified as a negative test result, where there is a significantly higher level of monthly loss in INL thickness, which may indicate accelerated neuroinflammation and CI. It must be noted that this diagnostic test was designed as a test of health, where a positive test result was associated with no CI.

These threshold values were used to sort data into a contingency table, which was analyzed by Fisher’s exact test. Basic diagnostic parameters such as sensitivity, specificity, positive predictive value (PPV), negative predictive value (NPV), diagnostic odds ratio (DOR), and likelihood ratio (LR) were calculated with GraphPad Prism 9.3.0.

#### Statistical evaluation of the diagnostic test

The efficacy of the diagnostic test was further appraised through the calculation of evaluative parameters listed in Supplementary Table S5.

## Supplementary Information

Supplementary Information.

## Data Availability

There were no additional data, code, and materials to provide.
