# Supplementary material for: Retinal inner nuclear layer thickness in the diagnosis of cognitive impairment explored using a C57BL/6J mouse model
Source: Sci Rep. 2023 May 19;13:8150. doi: 10.1038/s41598-023-35229-x (PMC10199094; doi:10.1038/s41598-023-35229-x)
Supplement: Supplementary file 1 — Supplementary Information. [file 41598_2023_35229_MOESM1_ESM.docx]

**Supplementary Information: Retinal inner nuclear layer thickness in the diagnosis of cognitive impairment explored using a C57BL/6J mouse model**

**Authors:**

Jack J. Maran^a^, Moradeke M. Adesina^b^, Colin R. Green^b^, Andrea Kwakowsky^c,d,e^, Odunayo O. Mugisho^a,b*^

**Affiliations:**

^a^Buchanan Ocular Therapeutics Unit, Department of Ophthalmology, New Zealand National Eye Centre, University of Auckland, New Zealand.

^b^Department of Ophthalmology, New Zealand National Eye Centre, University of Auckland, New Zealand.

^c^Centre for Brain Research, University of Auckland, New Zealand.

^d^Department of Anatomy and Medical Imaging, University of Auckland, New Zealand.

^e^Pharmacology and Therapeutics, School of Medicine, Galway Neuroscience Centre, National University of Ireland Galway, Ireland

***Corresponding Author:**

Odunayo O. Mugisho

Email address: lola.mugisho@auckland.ac.nz

Postal Address: Department of Ophthalmology, Faculty of Medical and Health Sciences, The University of Auckland, Private Bag 92019, Auckland, New Zealand.

**ORCiD identifiers:**

J. J. Maran ORCiD iD: 0000-0001-9366-4882

M. M. Adesina: 0000-0003-0315-805X

C.R. Green ORCID iD: 0000-0003-3459-6298

A. Kwakowsky ORCiD iD: 0000-0002-3801-4956

O.O Mugisho ORCiD iD: 0000-0002-4519-6727

# Supplementary Table S1

*Supplementary Table S1: Linear regression line equations for retinal layer thickness and discrimination index*

| **Layer** | **Equation** | **r^2^ value** | **Gradient significantly non-zero? (p-values)** |
| --- | --- | --- | --- |
| NFL-GCL-IPL | Y = 1.337x+ 48.22 | 0.02250 | 0.3826 |
| INL | Y = 3.812x+ 17.70 | 0.2475 | 0.0020** |
| ONL | Y = 5.657x+ 45.35 | 0.1367 | 0.0264* |
| TR | y= −1.847x+ 144.7 | 0.009172 | 0.5785 |

# Supplementary Table S2: Multiple linear regression of NFL-GCL-IPL, INL and ONL vs Total Retinal Thickness

*Table S2a: Multiple linear regression coefficients for TR thickness versus other retinal layers*

| **Layer** | **Regression Coefficient** | **P-value** | **VIF** | **R^2^ with other variables** |
| --- | --- | --- | --- | --- |
| Intercept | 99.07 | <0.0001**** | - | - |
| NFL-GCL-IPL | 1.257 | 0.0014** | 1.357 | 0.2630 |
| INL | -0.4865 | 0.2391 | 1.282 | 0.2201 |
| ONL | -0.1425 | 0.5368 | 1.618 | 0.3821 |

^Statistical analysis was carried out using GraphPad Prism 9.3.0 software. Data were analyzed by multiple linear regression. VIF: variance inflation factor. *p < 0.05; **p < 0.01.^

*Table S2b: Parameter covariance correlation matrix*

|  | **β0** | **β1** | **β2** | **β3** |
| --- | --- | --- | --- | --- |
| **β0** | 1.0000 |  |  |  |
| **β1** | -0.8408 | 1.0000 |  |  |
| **β2** | -0.4220 | 0.2628 | 1.0000 |  |
| **β3** | 0.1113 | -0.5122 | -0.4684 | 1.0000 |

^β denotes the coefficients of variables in the multiple linear regression equation. β0: intercept, β1: NFL-GCL-IPL, β2: INL, β3: ONL. Values in the table represent regression coefficients between variables.^

*Table S2c: Overall multiple regression model analysis of variance*

|  | **SS** | **DF** | **MS** | **F (DFn, DFd)** | **P-value** |
| --- | --- | --- | --- | --- | --- |
| **Regression** | 263.1 | 3 | 87.69 | F (3, 32) = 5.794 | P=0.0028** |
| **NFL-GCL-IPL** | 185.8 | 1 | 185.8 | F (1, 32) = 12.28 | P=0.0014** |
| **INL** | 21.78 | 1 | 21.78 | F (1, 32) = 1.439 | P=0.2391 |
| **ONL** | 5.899 | 1 | 5.899 | F (1, 32) = 0.3898 | P=0.5368 |
| **Residual** | 484.3 | 32 | 15.13 |  |  |
| **Total** | 747.3 | 35 |  |  |  |

SS: sum of squares, DF: degrees of freedom, MS: mean square, F: F-ratio, DFn: degrees of freedom (numerator), DFd: degrees of freedom (denominator).

# Supplementary Table S3: Multiple linear regression of the absolute change in NFL-GCL-IPL, INL and ONL vs the absolute monthly change in Total Retinal thickness

*Table S3a: Multiple linear regression coefficients for the absolute monthly change in TR thickness versus other retinal layers*

| **Layer** | **Regression Coefficient** | **P-value** | **VIF** | **R^2^ with other variables** |
| --- | --- | --- | --- | --- |
| Intercept | 1.5450 | 0.0126* | - | - |
| NFL-GCL-IPL | 1.5010 | 0.0007*** | 1.225 | 0.1839 |
| INL | 0.1047 | 0.8253 | 1.186 | 0.1565 |
| ONL | 0.1324 | 0.5655 | 1.053 | 0.05042 |

^Statistical analysis was carried out using GraphPad Prism 9.3.0 software. Data were analyzed by multiple linear regression. VIF: variance inflation factor. *p < 0.05; **p < 0.01, ***p < 0.001.^

*Table S3b: Parameter covariance correlation matrix*

|  | **β0** | **β1** | **β2** | **β3** |
| --- | --- | --- | --- | --- |
| **β0** | 1.0000 |  |  |  |
| **β1** | 0.2150 | 1.0000 |  |  |
| **β2** | 0.6273 | 0.3774 | 1.0000 |  |
| **β3** | 0.3934 | -0.1859 | 0.0465 | 1.0000 |

^β denotes the coefficients of variables in the multiple linear regression equation. β0: intercept, β1: NFL-GCL-IPL, β2: INL, β3: ONL. Values in the table represent regression coefficients between variables.^

*Table S3c: Overall multiple regression model analysis of variance*

|  | **SS** | **DF** | **MS** | **F (DFn, DFd)** | **P-value** |
| --- | --- | --- | --- | --- | --- |
| **Regression** | 73.28 | 3 | 24.43 | F (3, 20) = 6.812 | P=0.0024** |
| **NFL-GCL-IPL** | 57.75 | 1 | 57.75 | F (1, 20) = 16.10 | P=0.0007** |
| **INL** | 0.1794 | 1 | 0.1794 | F (1, 20) = 0.05003 | P=0.8253 |
| **ONL** | 1.224 | 1 | 1.224 | F (1, 20) = 0.3415 | P=0.5655 |
| **Residual** | 71.72 | 20 | 3.586 |  |  |
| **Total** | 145.0 | 23 |  |  |  |

SS: sum of squares, DF: degrees of freedom, MS: mean square, F: F-ratio, DFn: degrees of freedom (numerator), DFd: degrees of freedom (denominator). *p < 0.05; **p < 0.01, ***p < 0.001.

Supplementary Figure S4
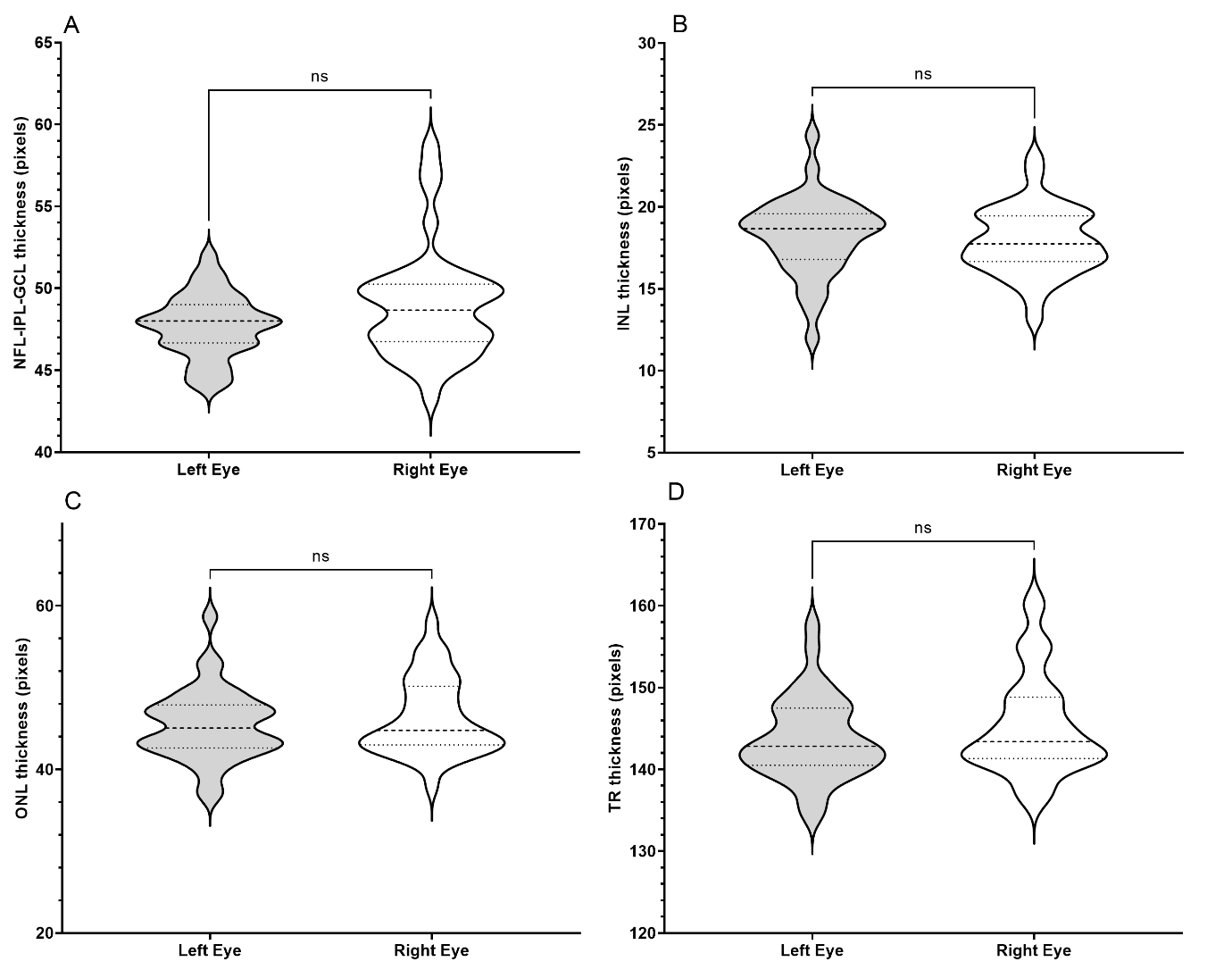


**Supplementary Figure S4:** Violin plots of retinal layer thickness in 4-month-old C57BL/6J mice at baseline, two months after baseline, and five months after baseline, grouped according to right or left eyes. Normality of residuals was also assessed by Anderson-Darling, D’Agostino-Pearson omnibus, Shapiro-Wilk, and Kolmogorov-Smirnov tests. Only INL data passed all normality and equal variance tests and were assessed for differences between groups with two-tailed unpaired t-tests. NFL-GCL-IPL, ONL and TR data failed all normality tests and were assessed for differences between groups with Mann-Whitney U tests. A) There is no significant difference between NFL-GCL-IPL thickness in left or right eyes (p = 0.1604). B) There is no significant difference between INL thickness between left or right eyes (p = 0.4939). C) There is no significant difference between ONL thickness between left or right eyes (p = 0.5850). D) There is no significant difference between TR thickness between left or right eyes (p = 0.3305). NFL-GCL-IPL = nerve fibre layer-ganglion cell layer-inner plexiform layer, INL = inner nuclear layer, ONL = outer nuclear layer, TR = total retina thickness.

Supplementary Table S5: Definitions for evaluative parameters of diagnostic tests

*Table S5: Evaluative parameters of diagnostic tests*

| **Parameter** | **Definition** | **Formula** |
| --- | --- | --- |
| Youden’s index (J) | An index that measures the performance of a dichotomous diagnostic test from 0 to 1 [75, 76]. | $J=sensitivity+specificity-1$ |
| Number needed to diagnose (NND) | The inverse of Youden’s index. The number of test subjects must be examined with the diagnostic test to correctly detect one person with cognitive impairment [76, 77]. | $NND=1/J$ |
| Number needed to misdiagnose (NNM) | The number of test subjects that need to be examined with the diagnostic test to detect one person with cognitive impairment incorrectly [76]. | $NNM=\frac{1}{1-accuracy}$ |
| Predictive Summary Index (Ψ or PSI) | The true total gain in predictive certainty obtained by performing a diagnostic test while considering population disease prevalence [76, 77]. | $\Psi=PPV+NPV-1$ |
| Number needed to predict (NNP) | The number of test subjects who need to be examined with the diagnostic test to correctly predict a diagnosis of cognitive impairment while considering the population disease prevalence [76, 77]. | $NNP=\frac{1}{\Psi}$ |
